# Multicomponent Pseudomonas aeruginosa Vaccines Eliciting Th17 Cells and Functional Antibody Responses Confer Enhanced Protection against Experimental Acute Pneumonia in Mice

**DOI:** 10.1128/iai.00203-22

**Published:** 2022-09-07

**Authors:** Mohammad Omar Faruk Shaikh, Matthew M. Schaefers, Christina Merakou, Marco DiBlasi, Sarah Bonney, Tiffany Liao, David Zurakowski, Margaret Kehl, David E. Tabor, Antonio DiGiandomenico, Gregory P. Priebe

**Affiliations:** a Division of Critical Care Medicine, Department of Anesthesiology, Critical Care and Pain Medicine, Boston Children’s Hospital, Boston, Massachusetts, USA; b Department of Anaesthesia, Harvard Medical School, Boston, Massachusetts, USA; c Department of Anesthesiology, Critical Care and Pain Medicine, Boston Children’s Hospital, Boston, Massachusetts, USA; d Vaccines and Immune Therapies, Biopharmaceuticals R&D, AstraZeneca, Gaithersburg, Maryland, USA; e Division of Infectious Diseases, Department of Pediatrics, Boston Children’s Hospital, Boston, Massachusetts, USA; University of Pennsylvania

**Keywords:** *Pseudomonas aeruginosa*, pneumonia, Th17, immunization, PopB, OprF/I

## Abstract

The Gram-negative pathogen Pseudomonas aeruginosa is a common cause of pneumonia in hospitalized patients. Its increasing antibiotic resistance and widespread occurrence present a pressing need for vaccines. We previously showed that a P. aeruginosa type III secretion system protein, PopB, elicits a strong Th17 response in mice after intranasal (IN) immunization and confers antibody-independent protection against pneumonia in mice. In the current study, we evaluated the immunogenicity and protective efficacy in mice of the combination of PopB (purified with its chaperone protein PcrH) and OprF/I, an outer membrane hybrid fusion protein, compared with immunization with the proteins individually either by the intranasal (IN) or subcutaneous (SC) routes. Our results show that after vaccination, a Th17 recall response from splenocytes was detected only in mice vaccinated with PopB/PcrH, either alone or in combination with OprF/I. Mice immunized with the combination of PopB/PcrH and OprF/I had enhanced protection in an acute lethal P. aeruginosa pneumonia model, regardless of vaccine route, compared with mice vaccinated with either alone or adjuvant control. Immunization generated IgG titers against the vaccine proteins and whole P. aeruginosa cells. Interestingly, none of these antisera had opsonophagocytic killing activity, but antisera from mice immunized with vaccines containing OprF/I, had the ability to block IFN-γ binding to OprF/I, a known virulence mechanism. Hence, vaccines combining PopB/PcrH with OprF/I that elicit functional antibodies lead to a broadly and potently protective vaccine against P. aeruginosa pulmonary infections.

## INTRODUCTION

The Gram-negative bacterium Pseudomonas aeruginosa causes a wide range of clinically important infections, mostly in hospitalized and immunocompromised patients, especially those requiring mechanical ventilation, those with burns or combat-related wounds, and in people with cystic fibrosis (CF). P. aeruginosa is the most common pathogen causing ventilator-associated pneumonia (VAP) worldwide, with a prevalence of 3% to 5% in adults ventilated for more than 48 h (1). The need for an effective vaccine for P. aeruginosa VAP is made even more urgent due to COVID-19, where the rate of VAP is strikingly high (2, 3). The steadily increasing antibiotic resistance encountered in P. aeruginosa clinical isolates (4–6), coupled with the relative dearth of new antibiotics in the pharmaceutical industry’s pipeline, also make paramount the need for new approaches to the development of an effective vaccine.

Although antibodies to the lipopolysaccharide (LPS) O antigen mediate high-level immunity to P. aeruginosa infections, vaccine strategies targeting the O antigen have not been successful to date and are stymied by O antigen variability and impaired immune responses when O antigens of different serotypes are combined (7–12). More recent P. aeruginosa vaccine strategies showing promise have focused on the outer membrane proteins OprF, OprI, and a OprF/I fusion protein (specifically OprF_190-342_-OprI_21-83_ called VC43, previously IC43), with and without flagellins (13–18). The OprF/I fusion protein vaccine VC43 remains a promising candidate as it induces multiple immune effectors in humans, including opsonic antibodies (18) and antibodies that inhibit IFN-γ binding to P. aeruginosa (19) (thereby interfering with a virulence mechanism [20]), and, via the OprF_329-342_ epitope, IFN-γ+T cell responses (15). However, despite positive results in a phase II trial demonstrating improved all-cause mortality (21), in a phase III trial targeting VAP prevention, VLA43 failed (22). We speculate that the failure of the VLA43 trial was related to the absence of an adjuvant and/or the lack of induction of a Th17 response, which our published (23, 24) studies show is critical for vaccine-induced broad protection.

Our previous work has identified PopB, a structural component of the type III secretion system (T3SS), as a protective T cell antigen that generates a Th17 response when administered intranasally with the Th17 adjuvant curdlan (23, 24). The *popB* gene has been found in nearly all P. aeruginosa strains (25, 26), and PopB is highly conserved and expressed during infection (25, 27, 28). A recombinant His-tagged version of PopB is soluble and stable only when copurified with its chaperone PcrH, so PopB is denoted PopB/PcrH. Our previous work has found that immunization with PcrH does not elicit a Th17 response and the addition of PopB is required for full protection (23, 24).

Here, we have studied the immune responses and protective efficacy against pneumonia in mice after vaccination with PopB/PcrH, OprF/I, or a mixture of both, via the intranasal (IN) or subcutaneous (SC) immunization routes. We report that the combined vaccine yields the highest protection regardless of route, likely due to eliciting both Th17 and functional antibody responses, particularly antibodies that inhibit the binding of OprF to IFN-γ.

## RESULTS

### Immunization of mice with PopB-containing vaccines elicits a Th17 response, while OprF/I does not.

**FIG 1:**
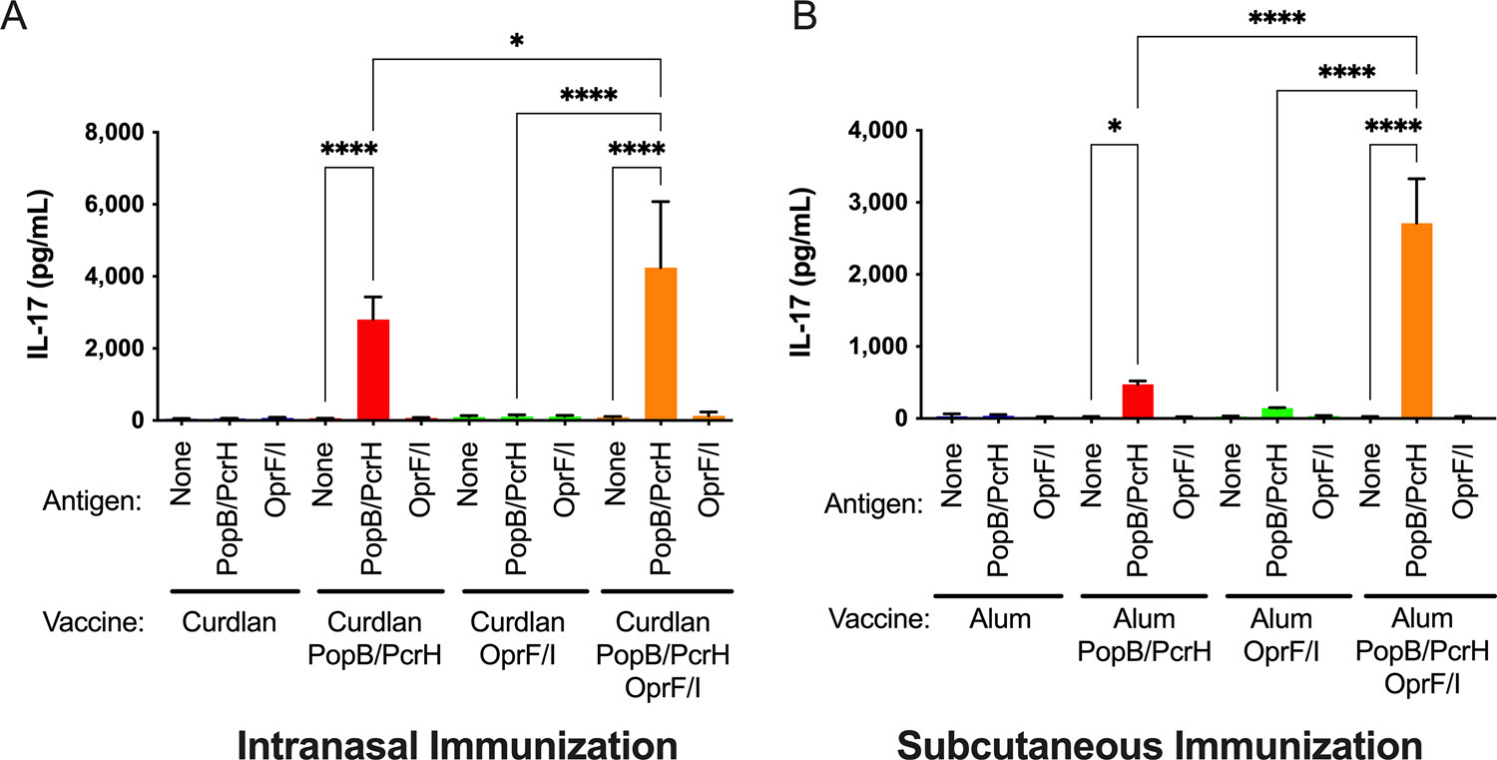
Vaccination of mice with PopB/PcrH but not OprF/I generates a Th17-response. Splenocytes from mice immunized either intranasally (A) or subcutaneously (B) with adjuvant alone (curdlan or Alum), adjuvant + 30 μg PopB/PcrH, adjuvant + 30 μg OprF/I, or adjuvant + 30 μg PopB/PcrH + 30 μg OprF/I were isolated 3 weeks after last immunization and stimulated with either PopB/PcrH, OprF/I or left unstimulated. IL-17 was measured by ELISA after 7 days of stimulation. Bars are the average of triplicate wells seeded with pooled splenocytes from four mice, and error bars are SDs, and are representative of immunizations, splenocyte isolation, and antigen stimulations conducted at least two times. * *p* of 0.0424 (A) or 0.0496 (B), **** denotes *P* < 0.0001 by one-way ANOVA followed by Šídák's multiple-comparison test compared with splenocytes stimulated with media only from mice within the same respective vaccine group or PopB/PcrH-stimulated splenocytes from immunization with PopB/PcrH or OprF/I alone to immunization with the PopB/PcrH + OprF/I combination.

With the failure of the phase III clinical trial using OprF/I as the vaccine antigen, novel approaches to vaccinate against P. aeruginosa infections are needed. Our previous work demonstrate that an effective Th17 response is required for robust protection against P. aeruginosa infections (23). We hypothesized the OprF/I vaccine used in the recent clinical trial was unable to induce a Th17 response, and potentially why it failed. To test this hypothesis, mice were immunized with PopB/PcrH, OprF/I, a combination of PopB/PcrH and OprF/I, or adjuvant alone. Our previous work with PopB has found that its chaperon, PcrH, is needed for effective expression and purification. Both proteins PopB and PcrH are used in immunization, but only PopB generates a Th17 response, and that response is protective (23, 24). Intranasal immunization has been previously described to induce Th17 recall responses that can be measured in the spleen (29). We prepared the recombinant fusion protein consisting of OprF_190-342_ and OprI_21-83_ as described for the OprF/I vaccine VLA43 (18). Mice were immunized IN using the Th17 adjuvant curdlan (30) or SC using aluminum hydroxide (Alum) as adjuvant. Three weeks after the last immunization, splenocytes were isolated and stimulated with either PopB/PcrH or OprF/I, and IL-17 was measured to quantify the Th17 response elicited by each vaccine. The immunization and challenge schedules are depicted in Fig. S1. Splenocytes from mice immunized with OprF/I did not produce IL-17 when stimulated with either OprF/I or PopB/PcrH when immunized via an IN route (Fig. 1A) or a SC route (Fig. 1B) demonstrating OprF/I does not induce a Th17 response in this mouse strain. Splenocytes isolated from mice immunized with PopB/PcrH, either alone or in combination with OprF/I produced IL-17 when stimulated with PopB/PcrH (Fig. 1). There were statistically significant (*P* value <0.05) increases in amounts of IL-17 produced in splenocytes isolated from mice immunized with the combination of PopB/PcrH and OprF/I compared with mice immunized with PopB/PcrH alone when immunized via IN or SC routes (~1.6-fold and ~5-fold, respectively) (Fig. 1) suggesting no evidence of inhibition or anergy when the OprF/I was mixed with PopB/PcrH. The increase in IL-17 when immunized with the combination of PopB/PcrH and OprF/I suggests a potential synergistic effect when the two antigens are used in combination.

### Immunization with the combination of PopB/PcrH and OprF/I induces significant protection against acute P. aeruginosa infection.

**FIG 2:**
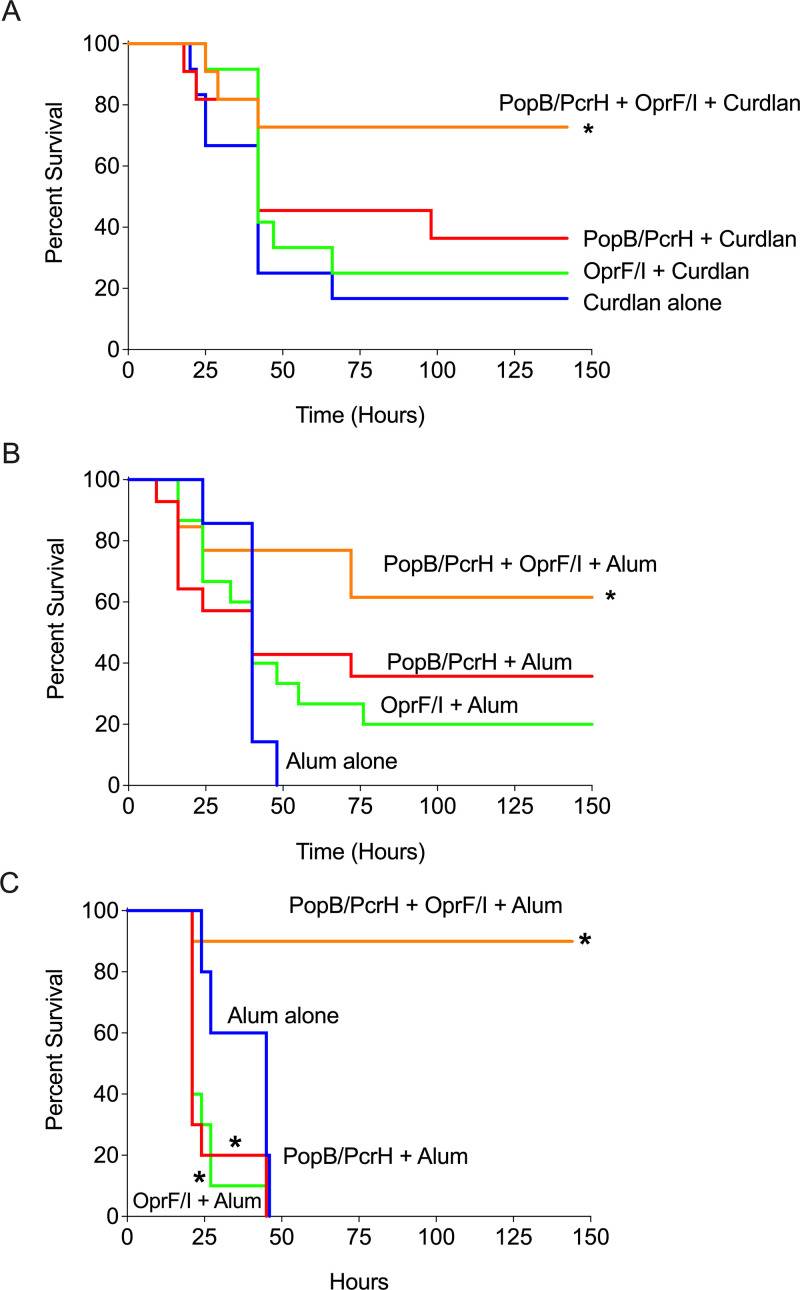
The combination of PopB/PcrH and OprF/I protects against acute lethal P. aeruginosa pneumonia. Mice immunized either intranasally (A) or subcutaneously (B and C) with adjuvant alone (curdlan or Alum), adjuvant + 30 μg PopB/PcrH, adjuvant + 30 μg OprF/I, or adjuvant + 30 μg PopB/PcrH + 30 μg OprF/I were intranasally challenged with P. aeruginosa strain N13 (2 × 10^6^ CFU/mouse) (A and B) or P. aeruginosa strain Exo^+^ PAO1 (8.1 × 10^5^ CFU) (C) 3 weeks after the last immunization. (C) Disease progression was monitored over 6 days, * denotes *P* value, 0.05 by log-rank test compared with adjuvant alone. *P* = 0.0132 (A); *P*= 0.0084 (B); *P* = 0.0002, 0.005, or 0.0091 for mice immunized with PopB/PcrH + OprF/I combination OprF/I alone, or PopB/PcrH alone, respectively (C). *n* = 11 to 12 mice per group, data are pooled from at least two independent experiments (A and B) and *n* = 10 mice per group from one experiment (C).

We next evaluated the protection provided by PopB/PcrH- and OprF/I-based vaccines against acute P. aeruginosa pneumonia after intranasal challenge. Three weeks after the final immunization mice were challenged with a lethal dose (>LD_100_) of P. aeruginosa strain N13 (2 × 10^6^ CFU/mouse), and the survival was monitored for 6 days. Mice immunized with the combination of PopB/PcrH and OprF/I showed significant protection against challenge compared with adjuvant alone in both the IN and SC routes (*P* values = 0.014 and 0.008, respectively, by log-rank test) (Fig. 2A and B). Mice immunized either IN or SC with PopB/PcrH or OprF/I alone were not significantly protected compared with adjuvant alone, demonstrating the combination of vaccine antigens conferred greater protection than either of the antigens alone. It is worth pointing out a higher dose of a different P. aeruginosa strain was used in our current study compared to our previously published findings using PopB/PcrH vaccines, which used the challenge strain ExoU^+^ PAO1, a highly virulent engineered strain expressing the ExoU cytotoxin and its chaperone by a plasmid (23, 24). We also challenged SC immunized mice with the highly virulent P. aeruginosa strain ExoU^+^ PAO1 (8.1 × 10^5^ CFU) and saw similar protection when mice were immunized with the combination of PopB/PcrH and OprF/I compared with mice immunized with the adjuvant alone (*P* value = 0.0002, by log-rank test) (Fig. 2C).

### Intranasal and subcutaneous immunization with PopB/PcrH or OprF/I elicit IgG responses.

**FIG 3:**
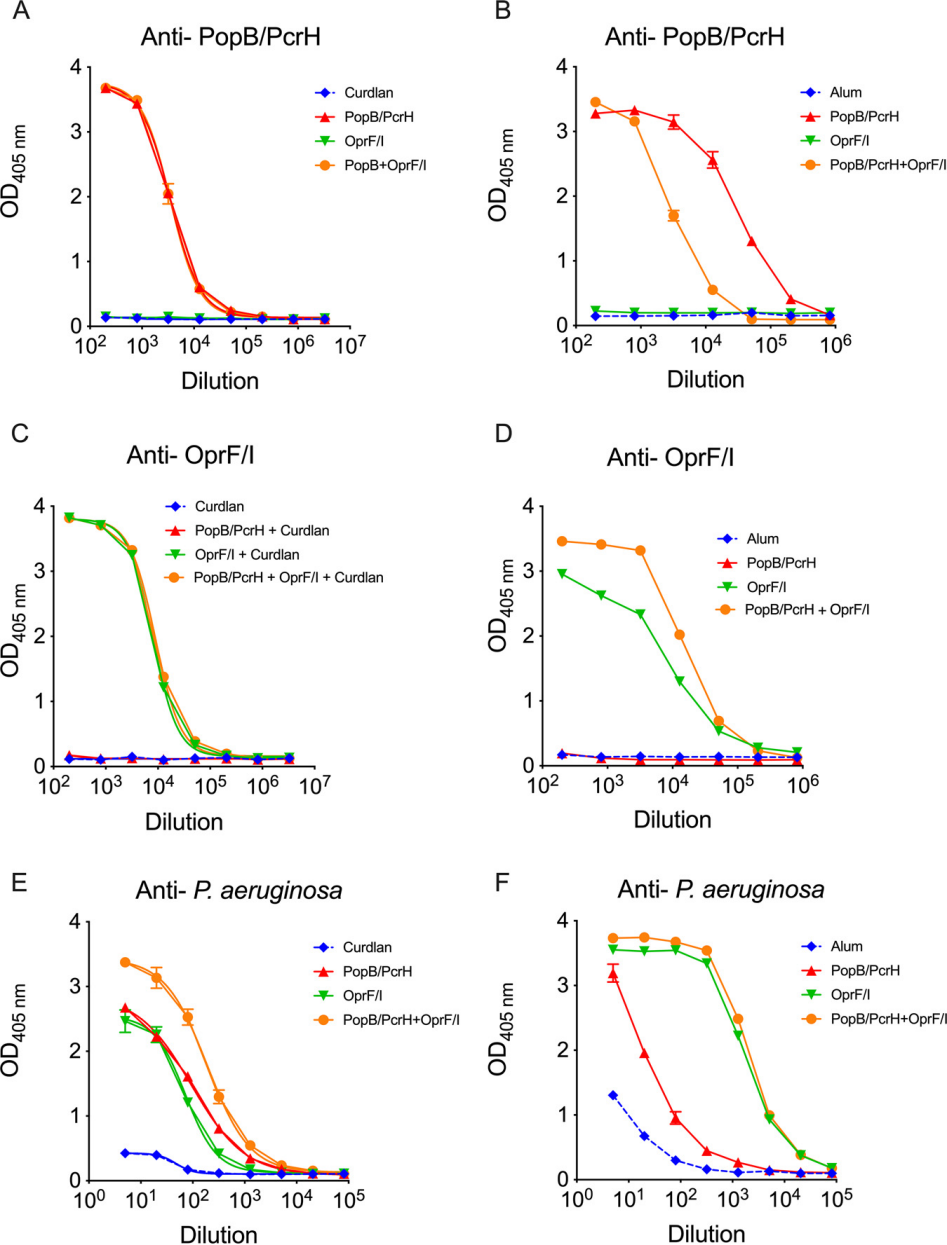
Vaccination with PopB/PcrH, OprF/I, or both, generate antigen-specific IgG responses that also recognize whole P. aeruginosa. Mice were immunized either intranasally (A, C, E) or subcutaneously (B, D, F) with adjuvant alone (curdlan or Alum), adjuvant + 30 μg PopB/PcrH, adjuvant + 30 μg OprF/I, or adjuvant + 30 μg PopB/PcrH + 30 μg OprF/I and sera were collected 3 weeks after the last immunization. Anti-PopB/PcrH (A, B), anti-OprF/I (C, D), and anti-whole P. aeruginosa strain N13 (E, F) IgG titers were measured using ELISA. Sera from three to four mice per group were pooled and measured in technical duplicates, and means are plotted with SD as error bars (error bars are smaller than symbol at many points). Data are representative of at least two independent immunization experiments.

To better describe the immune response generated by the various vaccines, we measured the humoral immune responses after immunization. Sera from immunized mice were collected 3 weeks after the third immunization, and IgG titers specific for PopB, OprF/I, and whole P. aeruginosa were measured (Fig. 3; Table S1A). As expected, mice generated IgG responses against the proteins they were immunized against. In IN-immunized mice, there was no difference in the EC_50_s of anti-PopB IgG titers in mice immunized with PopB/PcrH alone compared with a combination of PopB/PcrH and OprF/I, when comparing the 95% confidence intervals of EC_50_ determinations. Likewise, there was no difference in the anti-OprF/I titers in mice immunized with OprF/I alone compared with a combination of PopB/PcrH and OprF/I. Among SC-immunized mice, there was small, but statistically significant 1.7-fold increase in EC_50_s of anti-OprF/I IgG titers in mice immunized with the combination of PopB/PcrH and OprF/I compared with mice immunized with OprF/I alone. The biological significance of this small difference is unclear. Conversely, the anti-PopB titers were 10-fold lower in mice immunized with the combination of PopB/PcrH and OprF/I compared with the PopB/PcrH only group. We also measured the IgG titers against whole P. aeruginosa bacterial cells, using strain N13, which was the same strain used in challenge experiments. In IN-immunized mice, there was a significant ~2- or 2.7-fold increase in the EC_50_s of anti-P. aeruginosa IgG titers in mice immunized with the combination of PopB/PcrH and OprF/I compared with mice immunized with PopB/PcrH or OprF/I alone, respectively. In the SC-immunized mice, immunization with the combination of PopB/PcrH and OprF/I or OprF/I alone resulted in an ~200-fold significant increase of anti-P. aeruginosa IgG titers than the mice immunized with PopB/PcrH alone. We also measured the anti-PopB or anti-OprF/I titers of the IgG1 and IgG2c subclasses. In mice, a strong IgG1 response is associated with a Th2 response while a IgG2c response is associated with a Th1 response (31). Mice immunized via the SC route had higher IgG1 titers to both PopB and OprF/I suggesting a Th2 response (Fig. S2; Table S1B).

### Sera from mice immunized with OprF/I inhibit OprF binding to IFN-γ.

**FIG 4:**
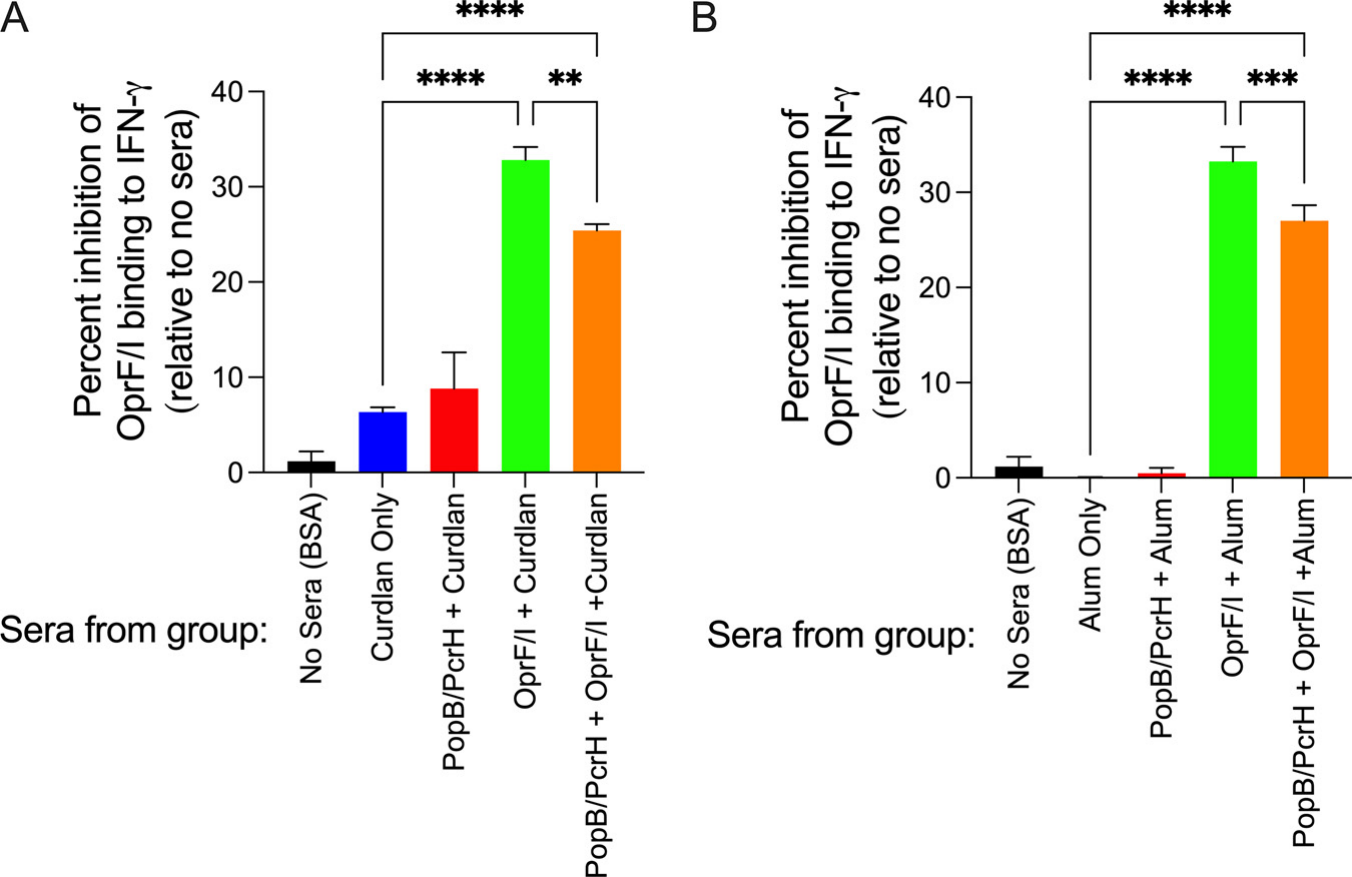
Sera from mice immunized with OprF/I inhibit binding of OprF/I to human IFN-γ. Sera were collected 3 weeks after the final immunization of mice immunized either intranasally (A) or subcutaneously (B) with adjuvant alone (curdlan or Alum), adjuvant + 30 μg PopB/PcrH, adjuvant + 30 μg OprF/I, or adjuvant + 30 μg PopB/PcrH + 30 μg OprF/I. Pooled sera were diluted 1:2 and assessed for inhibition of binding of human IFN-γ to OprF/I coated on wells of ELISA plates. IFN-γ bound to OprF/I was measured using anti-IFN-γ antibodies. The percent inhibition was calculated as the change in the amount of IFN-γ that bound OprF/I in the absence of sera. Sera from three to four mice per group were pooled and measured in triplicate, and means are plotted with SD as error bars. **** denotes *P* value <0.0001 by one-way ANOVA followed by the Šídák's multiple-comparison test. Data are representative of at least two independent experiments.

P. aeruginosa OprF can bind to human IFN-γ thereby enhancing virulence (20). Furthermore, anti-OprF/I antibodies are able to prevent the fusion protein OprF/I from binding to IFN-γ *in vitro*, which has been suggested as one mechanism that vaccination with OprF/I can prevent infection (19). Thus, we measured the ability of various sera to block OprF/I binding to human IFN-γ. Sera from mice immunized with OprF/I either alone or with PopB/PcrH had a statistically significant 25% to 33% reduction in OprF/I binding to IFN-γ when vaccinated either IN or SC (Fig. 4). Sera from mice immunized with adjuvant or PopB/PcrH alone did not inhibit OprF/I binding to IFN-γ. Surprisingly sera from OprF/I-immunized mice did not have opsonophagocytic killing (OPK) activity (Fig. S3) against target strain PAO1 (serotype O2/O5) and strain 9882-80 (serotype O11), perhaps due to using a different mouse strain compared with prior published work (32), Our previous results have shown that anti-PopB/PcrH antibodies do not mediate OPK (23, 24), and the current study confirmed those results (Fig. S3). Sera from mice immunized with vaccines containing PopB/PcrH and/or OprF/I were able to bind to whole P. aeruginosa strain PAO1 when measured by ELISA, where sera from IN-immunized mice showed a lower titer of antibodies against strain PAO1 compared with strain N13, but sera from SC-immunized mice had similar high titers against strains PAO1 and N13 (Fig. S4; Table S2).

## DISCUSSION

An effective vaccine to prevent P. aeruginosa infections remains an unrealized goal that could prevent a significant amount of morbidity and mortality (8, 12). The failure of recent clinical trials highlights the need for novel approaches. Our previous work demonstrated that IL-17 is critical in protection against LPS-heterologous strains of P. aeruginosa after vaccination with live-attenuated P. aeruginosa vaccines (32) and identified PopB as a protein stimulating Th17 responses and as a promising vaccine candidate for P. aeruginosa (23). In this study, we generated a novel vaccine that combined PopB/PcrH along with a recombinant fusion protein consisting of portions of OprF and OprI and evaluated vaccination via the IN and SC routes in mice using a combination of the antigens as well as the individual antigens.

We found that immunization with PopB-containing vaccines (either IN or SC) resulted in a Th17 response (Fig. 1). While OprF/I alone did not induce a Th17 response, there was a significant increase in the Th17 response when OprF/I was added to PopB/PcrH suggesting an adjuvant-like effect of OprF/I. OprI has been identified to function as an adjuvant that induces a Th2 response via TLR2 and TLR4 (33) and has been included in vaccine formulations for the prevention of viral and mycobacterial infections (34, 35). It is not known if OprI can serve as an adjuvant that can induce a Th17 response, or if OprI could be used as an adjuvant alone. Our ongoing work is investigating these questions. The Th17-responses to PopB are consistent with findings that both we and other groups have observed when immunizing with PopB-containing vaccines (23, 24, 36). Th17 responses are essential for host defense against a number of pathogens, including Salmonella enterica (37), Streptococcus pneumoniae (38), Klebsiella pneumoniae (39), Staphylococcus aureus (40, 41), Mycobacterium tuberculosis (42), and Candida albicans (43).

Vaccination with the combination of PopB/PcrH along with OprF/I resulted in significant protection against a lethal challenge of P. aeruginosa in our murine pneumonia model (Fig. 2). In the current study, we used a higher dose of P. aeruginosa strain N13, a clinical isolate of serotype O6 compared with our previous studies where we used P. aeruginosa strain PAO1 (serotype O2/O5) that expresses ExoU from a plasmid (23, 24). Immunization with either PopB/PcrH or OprF/I alone was not sufficient to protect mice against the lethal challenge doses used in this study; but the combination vaccine protected mice, whether vaccinated IN or SC. It is worth pointing out that a different dose of a different P. aeruginosa strain was used in challenge experiments in the current study compared with our published studies using PopB-based vaccines (23, 24). Future work is needed to better understand the differences in protection when challenged with different doses of different strains in addition to different disease models. Our previous work has identified that the Th17 response is required for broad serotype-heterologous protection against P. aeruginosa infection (23), and as such, Th17 responses are likely contributing to the protection observed in the current study. The total amount of protein used in the different vaccine groups varied depending on whether one or two antigens were used and may have contributed to the differing immune responses observed between the single and combination antigen vaccinations.

Immunization with PopB/PcrH, OprF/I, or the combination of the two proteins elicited antigen-specific IgG antibody responses. The immunizations also elicited an antibody response that was able to recognize whole P. aeruginosa coated on ELISA plates. The contributions to protection of these antibodies remains unclear. Our previous work, and current work with PopB-based immunization, demonstrate that antibodies generated in response to PopB do not have OPK activity (Fig. S3) (23, 24). Studies in humans (18), nonhuman primates (44), and other mouse strains (14) found that immunization with OprF/I generates antibodies with OPK activity, but we observed no such activity in the current study even though antibodies from mice immunized with PopB/PcrH and/or OprF/I-containing vaccines were able to bind the target strain PAO1 by ELISA (Fig. S4). The overall response to the OprF/I was IgG1 biased suggesting a Th2 response, which is also consistent with a previous study (14). We did find that mice immunized with OprF/I generated antibodies that were able to block the binding of OprF/I to IFN-γ, a host-sensing mechanism shown to enhance the virulence of P. aeruginosa (20). These antibodies that prevent the OprF-IFN-γ interaction are predicted to be one mechanism that immunization with OprF/I can reduce P. aeruginosa virulence (19).

Based on the failure of the OprF/I vaccine (VLA43) in phase III clinical trials, an effective vaccine that can prevent P. aeruginosa infections will need to elicit multiple mechanism of action, with Th17 responses as a critical component. The current work suggests that combining PopB with OprF/I significantly improves protective efficacy against acute lethal pneumonia in mice compared with either protein alone, advancing us one step closer to a broadly and potently protective vaccine for P. aeruginosa.

## MATERIALS AND METHODS

### Generation of protein expression vectors.

A gene fragment encoding Escherichia coli*-*codon-optimized OprF_190-342_ and OprI_21-83_ was synthesized with a 5 prime *ndeI* and a 3 prime *xhoI* restriction site (Genescript). This fragment was then cloned into the *ndeI* and *xhoI* site of pET24a(+) creating pET24a(+)-OPRF/I. Insert was confirmed by Sanger sequencing. The pET28b-based PopB/PcrH expressing vector was previously described (23).

### Expression and purification of OprF/I and PopB/PcrH from E. coli.

Purification of OprF/I was performed as previously described in patent filings (https://patents.google.com/patent/EP2686339A1/). Briefly, E. coli BL21(DE3) carrying pET24a(+)-OPRF/I was grown in 2 L of fresh LB media with kanamycin and grown at 37°C. When an OD_600_ of 0.8 was reached, the culture was induced with 1 mM IPTG (isopropyl β-d-1-thiogalactopyranoside) and incubated for an additional 3.5 h. E. coli cells were then harvested using centrifugation for 10 min at 7,800 g in 4°C. Cells were resuspended in lysis buffer (buffer A) containing 8 M urea, 20 mM Tris-HCl, 100 mM KCl, 200 mM NaCl, and 10 mM imidazole and then sonicated. Lysates of cells were centrifuged at 10,000 g for 30 min and supernatants were purified through an IMAC column. Column resin was washed with 50 column volumes of buffer A containing 0.1% Triton X-114 followed by 20 column volumes of buffer A. OprF/I was then eluted in buffer A containing 250 mM imidazole. Fractions of elution underwent refolding dialysis with urea, endotoxin removal with polylysine resin, and then final dialysis with reoxidation of the purified material using 1 mM dithiothreitol (DTT). PopB/PcrH was purified as previously described (23).

### Immunization of mice and murine pneumonia model.

All animal protocols and procedures were approved by the Boston Children’s Hospital Institutional Animal Care and Use Committee (assurance number A3303-01). The specific protocol numbers are 18-01-3617R and 20-12-4326R. All animal protocols are compliant with NIH Office of Laboratory Animal Welfare, Guide for the Care and Use of Laboratory Animals, the U.S. Animal Welfare Act, and PHS Policy on Humane Care and Use of Laboratory Animals. Friend virus B NIH Jackson (FVB/N) mice (6 to 8 weeks old, female) were obtained from the Jackson Laboratories and maintained in-house for both vaccination periods and challenge experiments. All immunization experiments were conducted at least two times. In these experiments ~15 mice per group were immunized, three to four mice were used for splenocyte isolation and serum collection, while the rest of the mice were used in challenge experiments. The immunization and challenge schedule is depicted in Fig. S1.

**(i) Vaccine formulation and immunization.** SC formulated vaccines contained in 200 μL per dose of the following: 30 μg of each protein (PopB/PcrH, OprF/I, or both) mixed with Alum (Alhydrogel; aluminum hydroxide adjuvant 2%, 25 μL, 250 μg/dose; InvivoGen) and suspended in saline with incubated for 1 h at room temperature with gentle rotation for adsorption prior to vaccination. SC vaccination was conducted once every 2 weeks for a total of three vaccinations (day 0, 14, and 28) with Alum alone as negative control (31). Vaccines formulated for IN immunization contained in 30 μL per dose (15 μL per nostril) the following: 30 μg of each protein (PopB/PcrH, OprF/I, or both) mixed with curdlan (150 μg/dose) (Sigma-Aldrich) and suspended in PBS. Vaccination was conducted once every week for a total of three vaccinations (day 0, 7, and 14) with curdlan alone as negative control (32).

**(ii) Mouse sera collection.** Blood samples from mice were collected retro-orbitally 3 weeks after the third vaccination (i.e., day 35 [for IN] and day 56 [for SC], where day 0 is the first vaccine dose). The sera were separated using serum separator tubes (BD). Sera were aliquoted and stored at −80°C until use.

**(iii) Challenge experiments.** Mice were challenged with P. aeruginosa using previous published methods (23, 24). Briefly, P. aeruginosa strain N13 was grown overnight from a frozen stock at 37°C on tryptic soy agar (TSA) plates. Bacteria scraped from the plate were suspended in 10 mL PBS (Invitrogen) to OD_650nm_ of 0.55, which corresponds to approximately 10^9^ CFU/mL. P. aeruginosa strain Exo^+^ PAO1 was grown on TSA plates containing 400 μg/mL of carbenicillin and adjusted to an OD_650nm_ of 0.45. Bacterial suspensions were diluted to prepare the inocula, and bacterial doses were confirmed by serial dilution and plating. Mice were anesthetized with ketamine/xylazine, and intranasal inoculation was performed by the administration of 20 μL (2 × 10^6^ CFU/dose) of the inoculum, applying 10 μL into each nare. Mice were monitored for 6 days, and moribund mice were euthanized (31).

### Opsonophagocytic killing assays.

OPK assays were performed following methods as previously described (45). While OPK activity below 50% can be statistically significant, OPK activity is generally only biologically significant when above 50% (24).

### Splenocyte isolation and coculture assays for IL-17 secretion.

Splenocytes were isolated and stimulated with vaccine antigens as previously described (23). Briefly, spleens were aseptically removed and suspended in PBS containing 2% heat-inactivated FCS. Spleens were disaggregated by passing through 100-micron nylon screens into a petri dish. Erythrocytes were lysed using a Mouse Erythrocyte Lyse Kit (R&D Systems) per the manufacturer’s protocol. Cells were centrifuged and resuspended in 5 mL cR10 (RPMI with 2 mM glutamine, 1 mM sodium pyruvate, 1× nonessential amino acids solution, 1× penicillin/steptomycin, 55 μM 2-mercaptoethanol, and 10% heat-inactivated FCS, all from Invitrogen), adjusted 1 × 10^6^ cells/mL, and seeded into 96-well round-bottom polystyrene plates. Cells were stimulated with 1 μg/mL protein for 7 days at 37°C in 5% CO_2_. Supernatants were collected and assayed for IL-17 by ELISA (R&D Systems).

### ELISAs.

ELISAs to measure PopB-, OprF/I-, or whole P. aeruginosa*-s*pecific IgG titers were performed with plates (Immulon 4HBX) coated with proteins at 1 μg/mL or live bacteria, as previously described (32). P. aeruginosa strain N13 was grown in LB containing 5 mM EGTA to induce the type III secretion system (TTSS) to maximize PopB expression (46).

### OprF/I-IFN-γ binding inhibition assays.

Assays for the inhibition of binding of human IFN-γ to plate-bound OprF/I was performed as previously reported (19). Briefly, 100 μL of OprF/I (1 μg/mL) was applied to the wells of an ELISA plate (Immulon 4HBX) and incubated overnight at 4°C. After washing with PBST, the plates were blocked with 5% BSA in PBS for 1 h at 37°C. OprF/I-coated plates were then incubated with 100 μL of 1:2 dilutions of antisera for 2 h at 37°C. After washing, 100 μL of human IFN-γ (0.5 μg/mL) (R & D Systems) was added to the wells and incubated overnight at 4°C. The bound IFN-γ was detected using the detection antibody and substrate system using human IFN-γ DuoSet reagents (R & D Systems) according to the manufacturer’s protocol.

### Statistical analyses.

All analyses were performed using Prism (GraphPad Software). Survival data were analyzed with the Kaplan-Meier method and log-rank tests. Parametric data were analyzed by ANOVA with Šídák's *post hoc* multiple-comparison test for pairwise comparisons between groups. EC_50_ was determined by nonlinear regression with 95% confidence intervals based on profile likelihood. We determined EC_50_ values to be statistically significant if the 95% CIs did not overlap.
