# Supplementary material for: Multicomponent Pseudomonas aeruginosa Vaccines Eliciting Th17 Cells and Functional Antibody Responses Confer Enhanced Protection against Experimental Acute Pneumonia in Mice
Source: Infect Immun. 2022 Sep 7;90(10):e00203-22. doi: 10.1128/iai.00203-22 (PMC9584304; doi:10.1128/iai.00203-22)
Supplement: Supplemental file 1 — Fig. S1 to S4 and Tables S1 and S2. Download iai.00203-22-s0001.pdf, PDF file, 1.1 MB [file iai.00203-22-s0001.pdf]

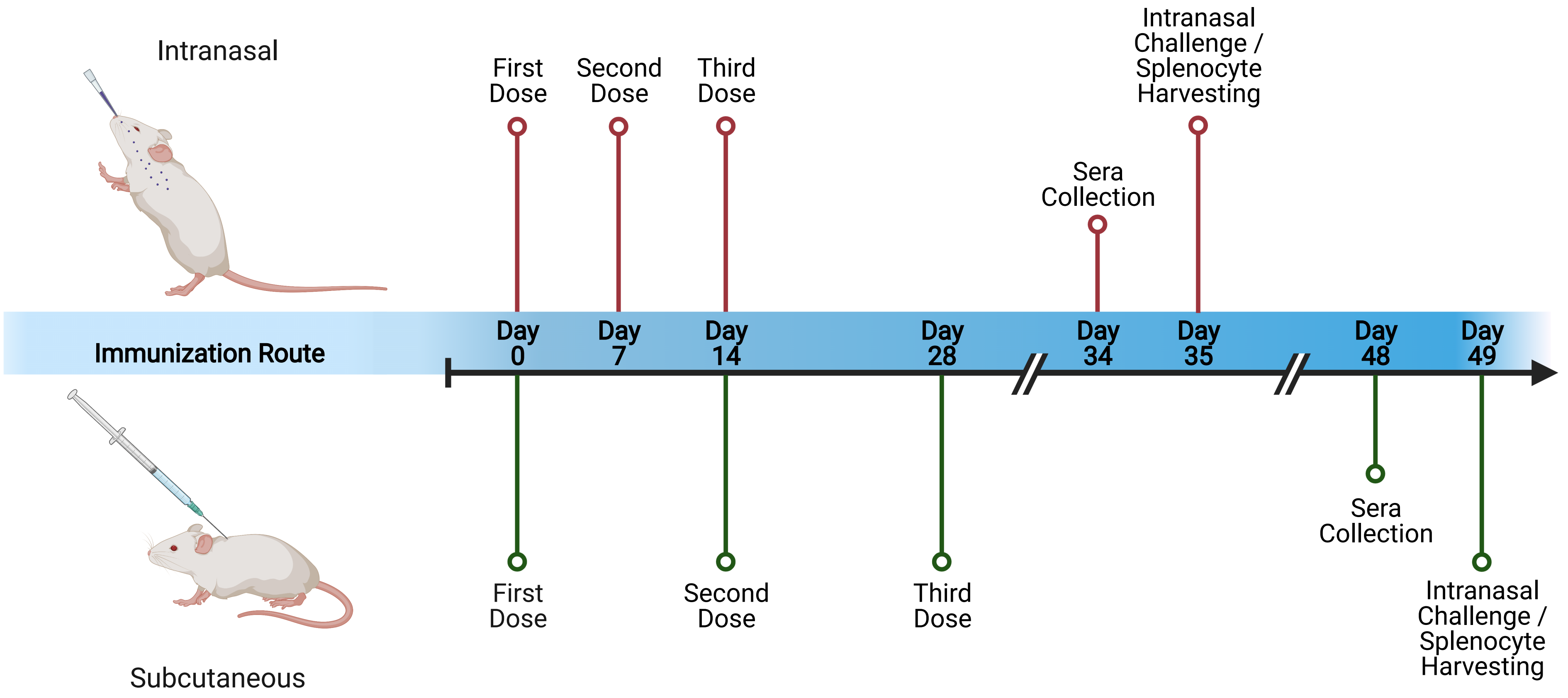
Intranasal
First Dose
Second Dose
Third Dose
Sera Collection
Intranasal Challenge / Splenocyte Harvesting
Immunization Route
Day 0
Day 7
Day 14
Day 28
Day 34
Day 35
Day 48
Day 49
First Dose
Second Dose
Third Dose
Sera Collection
Intranasal Challenge / Splenocyte Harvesting
Subcutaneous

Figure S1

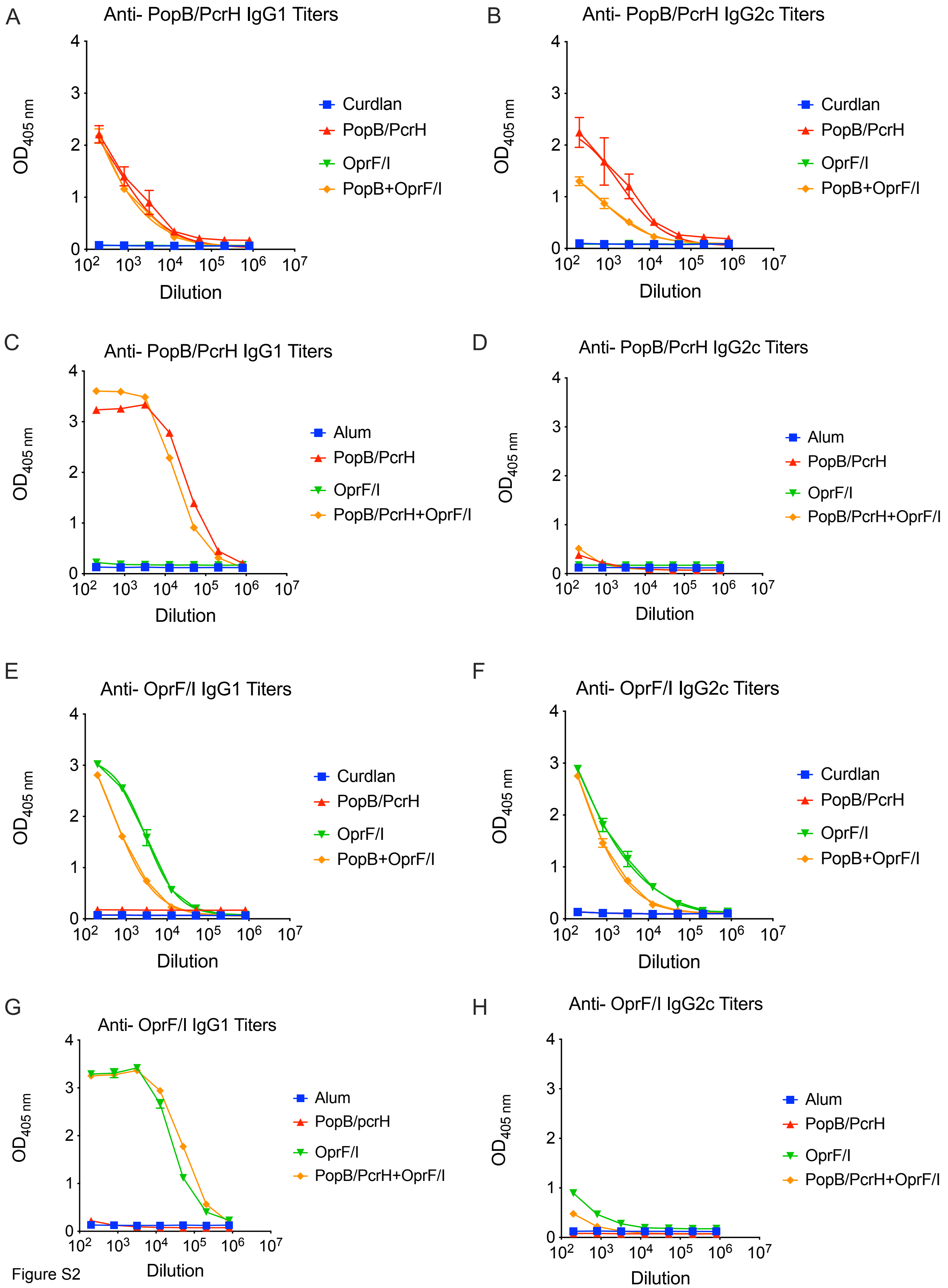
A
Anti- PopB/PcrH IgG1 Titers
B
Anti- PopB/PcrH IgG2c Titers
Curdlan
PopB/PcrH
OprF/I
PopB+OprF/I
C
Anti- PopB/PcrH IgG1 Titers
D
Anti- PopB/PcrH IgG2c Titers
Alum
PopB/PcrH
OprF/I
PopB/PcrH+OprF/I
E
Anti- OprF/I IgG1 Titers
F
Anti- OprF/I IgG2c Titers
G
Anti- OprF/I IgG1 Titers
PopB/pcrH
H
Anti- OprF/I IgG2c Titers
$OD_{405\ nm}$
Dilution
0
1
2
3
4
10^2
10^3
10^4
10^5
10^6
10^7

Figure S2

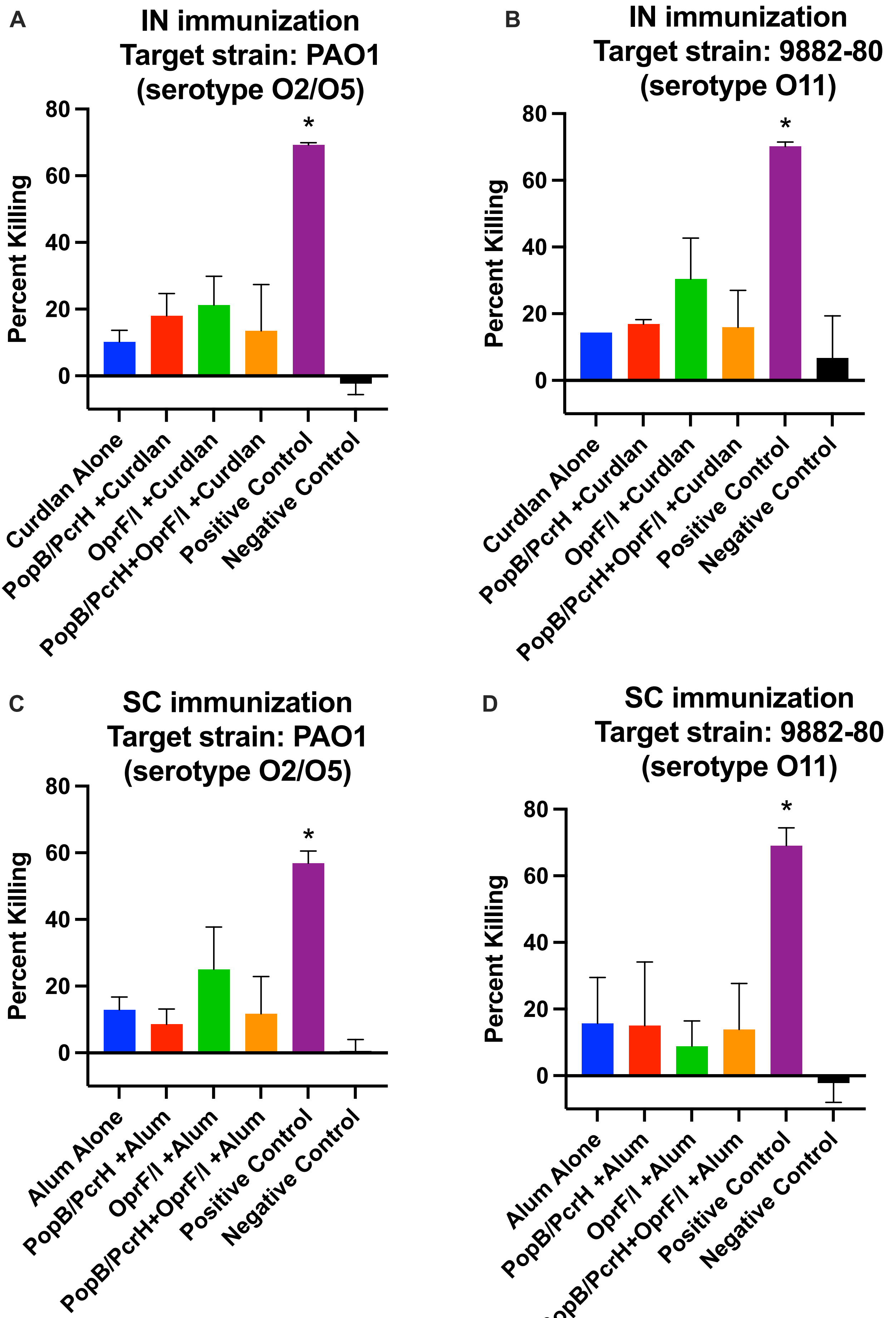
A
IN immunization
Target strain: PAO1
(serotype O2/O5)
Percent Killing
80
60
40
20
0
*
Curdlan Alone
PopB/PcrH +Curdlan
OprF/I +Curdlan
PopB/PcrH+OprF/I +Curdlan
Positive Control
Negative Control
B
IN immunization
Target strain: 9882-80
(serotype O11)
Percent Killing
80
60
40
20
0
*
Curdlan Alone
PopB/PcrH +Curdlan
OprF/I +Curdlan
PopB/PcrH+OprF/I +Curdlan
Positive Control
Negative Control
C
SC immunization
Target strain: PAO1
(serotype O2/O5)
Percent Killing
80
60
40
20
0
*
Alum Alone
PopB/PcrH +Alum
OprF/I +Alum
PopB/PcrH+OprF/I +Alum
Positive Control
Negative Control
D
SC immunization
Target strain: 9882-80
(serotype O11)
Percent Killing
80
60
40
20
0
*
Alum Alone
PopB/PcrH +Alum
OprF/I +Alum
PopB/PcrH+OprF/I +Alum
Positive Control
Negative Control

Figure S3

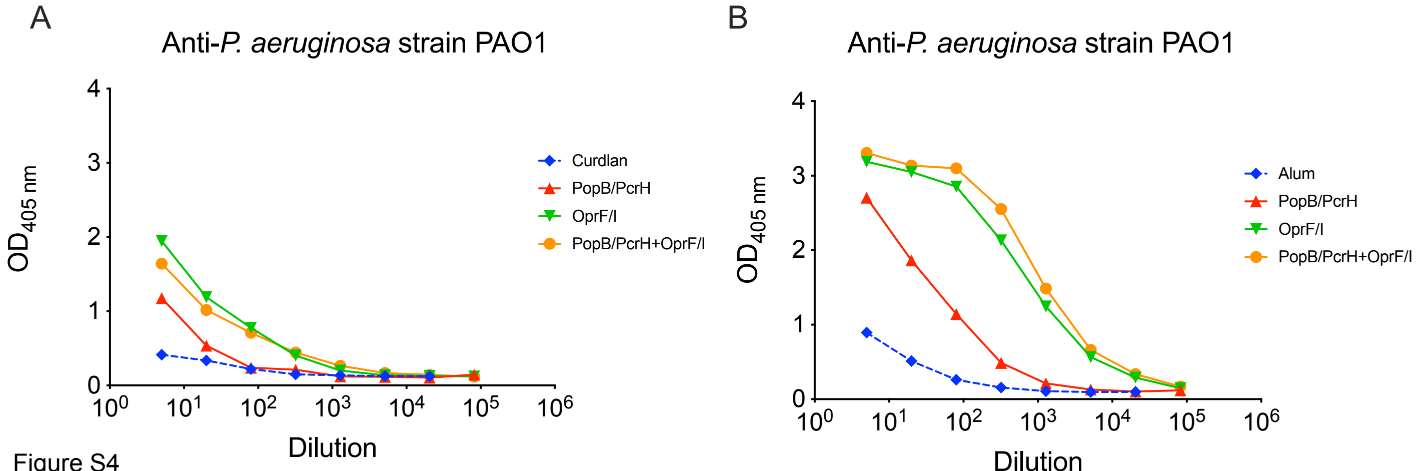
A
Anti-P. aeruginosa strain PAO1
OD405 nm
Curdlan
PopB/PcrH
OprF/I
PopB/PcrH+OprF/I
Dilution
B
Anti-P. aeruginosa strain PAO1
OD405 nm
Alum
PopB/PcrH
OprF/I
PopB/PcrH+OprF/I
Dilution

Figure S4

Supplemental Figure Legends

Figure S1. The immunization and challenge schedule of mice immunized by the intranasal or subcutaneous route. Created with BioRender.com

Figure S2. Vaccination with PopB/PcrH, OprF/I, or both, generates antigen-specific IgG1 and IgG2c responses. Mice were immunized either intranasally (A,B,E,F) or subcutaneously (C,D,G,H) with adjuvant alone (curdlan or Alum), adjuvant + 30 µg PopB/PcrH, adjuvant + 30 µg OprF/I, or adjuvant + 30 µg PopB/PcrH + 30 µg OprF/I, and sera were collected three weeks after the last immunization. Anti-PopB/PcrH (A,C) and anti-OprF/I (E,G) IgG1 titers and anti-PopB/PcrH (B,D) and anti-OprF/I (F,H) IgG2c titers were measured using ELISA. Sera from 3-4 mice per group were pooled and measured in duplicate, and means are plotted with SD as error bars (error bars are smaller than symbol at many points). Data are representative of at least two independent experiments.

Figure S3. Vaccination with PopB/PcrH, OprF/I, or both, do not generate significant opsonophagocytic killing activity. Mice were immunized either intranasally (A,B) or subcutaneously (C,D) with adjuvant alone (curdlan or Alum), adjuvant + 30 µg PopB/PcrH, adjuvant + 30 µg OprF/I, or adjuvant + 30 µg PopB/PcrH + 30 µg OprF/I, and sera were collected three weeks after the last immunization. *P aeruginosa* strain PAO1 (serotype O2/O5) and strain 9882-80 (serotype O11) were target strains, and sera were used at 1:30 dilution. As a positive control, an anti-Psl monoclonal antibody Cam003 (10µg/ml) was used. R347, human monoclonal antibody to HIV gp120 (10µg/ml) was used as a negative control. *denotes $p<0.05$ by one-way

ANOVA followed by Dunnett's *post-hoc* multiple comparison test when compared to the adjuvant only vaccine group (curdlan or Alum).

Figure S4. Vaccination with PopB/PcrH, OprF/I, or both, generate an IgG response that also recognizes whole cells of *P. aeruginosa* strain PAO1. Mice were immunized either intranasally (A) or subcutaneously (B) with adjuvant alone (curdlan or Alum), adjuvant + 30 µg PopB/PcrH, adjuvant + 30 µg OprF/I, or adjuvant + 30 µg PopB/PcrH + 30 µg OprF/I and sera were collected three weeks after the last immunization. Anti- whole *P. aeruginosa* strain PAO1 IgG titers were measured using ELISA. Sera from 3-4 mice per group were pooled and measured in technical duplicates, and means are plotted with SD as error bars (error bars are smaller than symbols at many points).

Table S1. EC50 calculations for titers measured by ELISA.

A. Total IgG:

	Vaccine Groups	Anti-PopB/PcrH IgG		Anti-OprF/I IgG		Anti- *P. aeruginosa* IgG	
		EC_{50}	95% CI	EC_{50}	95% CI	EC_{50}	95% CI
SC	Alum	NC	NC	NC	NC	NC	NC
	PopB/PcrH	33,683	29,877 - 38,103	NC	NC	10	4 - 17
	OprF/I	NC	NC	9,330	7,760 - 11,133	1,832	1,667 - 2,019
	PopB/PcrH + OprF/I	3,007	2,718 - 3,313	15,699	14,107 - 17,549	2,042	1,911 - 2,184
IN	Curdlan	NC	NC	NC	NC	NC	NC
	PopB/PcrH	3,527	3,340 - 3,721	NC	NC	94	82 - 107
	OprF/I	NC	NC	7,992	7,616 - 8,381	72	61 - 83
	PopB/PcrH + OprF/I	3,499	3,244 - 3,770	8,856	8,326 - 9,407	190	163 - 220

B. IgG Subclasses:

	Vaccine Groups	Anti-PopB/PcrH IgG1		Anti-OprF/I IgG1		Anti-PopB/PcrH IgG2c		Anti-OprF/I IgG2c	
		EC_{50}	95% CI	EC_{50}	95% CI	EC_{50}	95% CI	EC_{50}	95% CI
SC	Alum	NC	NC	NC	NC	NC	NC	NC	NC
	PopB/PcrH	37,957	34,291 - 42,045	NC	NC	274	13 - 572	NC	NC
	OprF/I	NC	NC	28,524	24,859 - 32,700	ND	NC	113	NC
	PopB/PcrH + OprF/I	18,829	16,762 - 21,227	53,868	48,270 - 60,591	3.3	NC	0.4	NC
IN	Curdlan	NC	NC	NC	NC	NC	NC	NC	NC
	PopB/PcrH	526	NC	NC	NC	1,974	NC	NC	NC
	OprF/I	NC	NC	3,013	2,593 - 3,449	NC	NC	96	NC
	PopB/PcrH + OprF/I	69	NC	472	312 - 623	722	49 - 1,429	192	13 - 443

Abbreviations:

NC= not calculated; EC_{50}= half maximal effective concentration; 95% CI = 95% of confidence interval

Table S2. EC50 calculations for titers measured by ELISA.

	Vaccine Groups	Anti- *P. aeruginosa* strain PAO1 IgG	
		**EC_{50}**	**95% CI**
SC	Alum	7	0.8-14
	PopB/PcrH	24	12-34
	OprF/I	670	602-745
	PopB/PcrH + OprF/I	982	876-1103
IN	Curdlan	37	5-65
	PopB/PcrH	NC	NC
	OprF/I	3	0.1-14
	PopB/PcrH + OprF/I	NC	NC

Abbreviations:

NC= not calculated; EC_{50}= half maximal effective concentration; 95% CI = 95% of confidence interval
